# Behavior plan and the intention–behavior gap in physical activity among adolescents and college students: a systematic review and meta-analysis

**DOI:** 10.3389/fpubh.2026.1950356

**Published:** 2026-09-24

**Authors:** Yao Yin, Xiaodong Sun, Jing He, Yufei Qi, Guisen Li

**Affiliations:** 1 School of General Education and Faculty of Physical Education, Beijing College of Finance and Commerce, Beijing, China; 2 Department of Physical Education, Beijing University of Chinese Medicine, Beijing, China; 3 Department of Physical Education, Central South University, Changsha, Hunan, China; 4 Department of Physical Education, Peking University, Beijing, China

**Keywords:** extended theory of planned behavior, meta-analytic, physical activity, structural equation modeling, the moderating factors

## Abstract

**Objectives:**

(1) To conduct a meta-analysis on the structure of the TPB extended model to examine the predictive effects of its components on physical activity participation among adolescents and college students; (2) To investigate the moderating effects of key research dimensions, including participant groups, cultural backgrounds, physical activity measurement methods, study designs, and gender composition (proportion of females), on the relationships between variables in the TPB extended model and behavioral models for adolescents and college students physical activity; (3) To test the mediating effects of the TPB extended model; (4) To examine the direct effect of perceived behavioral control on adolescents’ and college students’ behavior.

**Methods:**

A systematic literature search was conducted across multiple databases to identify studies applying the extended Theory of Planned Behavior (TPB) model to physical activity (PA) in adolescents and college students (approximately 10–24 years). A total of 28 articles (including 31 independent samples, with a total of 19,652 unique participants) met the inclusion criteria. Pairwise meta-analyses were performed using random-effects models in CMA 3.0 software. Meta-analytic structural equation modeling (MASEM) was conducted in Amos 24.0, with covariance matrix and harmonic mean constructed based on the summarized correlation matrix. Moderation analyses were conducted to examine the moderating effects of participant group, cultural background, physical activity measurement method, study design, and gender composition. Publication bias was assessed using funnel plots and Egger’s regression tests, and methodological quality was evaluated using the adapted Newcastle-Ottawa Scale (NOS-xs).

**Results:**

The TPB extended model explained 54.7% of intention differences, 29.2% of behavior differences, and 28.9% of behavior plan differences. Behavioral intention was found to be a significant statistical mediator variable connecting the three major factors of TPB with behavior, and additional indirect effects have been observed through the sequential pathway of behavioral intention and behavior plan. Moderator analyses revealed that the strength of several TPB relationships was contingent upon participant characteristics (adolescents and college students), cultural background, measurement methods, and study design.

**Conclusion:**

The TPB extended model demonstrates predictive utility for adolescents and college student’s physical activities at the aggregate level and can explain, on average, substantial variations in behavioral intention, behavior, and behavior plan across the diverse studies synthesized here. Nevertheless, the extreme heterogeneity (*I*^2^ > 90% for many pairwise associations) indicates that effect sizes vary considerably across populations, instruments, and study designs, limiting the generalizability of any single pooled estimate to specific subgroups or settings. In this cross-sectional synthesis, Perceived Behavioral Control (PBC) showed the strongest statistical association with behavioral intention. Behavior plan was also statistically associated with the relationship between behavioral intention and action, and may represent a potential explanatory pathway; however, the predominantly cross-sectional design precludes any causal or temporal inferences. The action path of this model is moderated by participant group, cultural background, measurement method, and study design, highlighting its contextual sensitivity. These findings underscore that PBC and behavior plan may be key variables for inclusion in future intervention designs, although their causal impact on behavior change still remains to be established via controlled trials.

## Introduction

Promoting long-term participation in physical activity (PA) is a critical research focus in the field of public health. Regular physical activity can bring substantial benefits, including improving cardiovascular health, enhancing cognitive function, and reducing the risk of chronic diseases such as obesity and diabetes (1–3). It also plays a positive role in alleviating anxiety and depression (4–6). The WHO defines “youth” as the population aged 15 to 24 (7), which currently accounts for 16% of the global population, approximately 1.2 billion people, and is expected to grow to nearly 1.3 billion by 2030. However, in the physical activity literature, the terms “adolescent” and “youth” are often used interchangeably, and the World Health Organization defines adolescence as 10–19 years. To capture the full breadth of relevant empirical evidence on physical activity among school-aged and college populations, this review adopts a broader operational definition encompassing both adolescents (including middle and high school students) and college students, thereby covering a developmental continuum from approximately 10 to 24 years. Engaging in physical activities during this life stage is not only a means to acquire health benefits and reduce health risks, but also holds significant implications for broader societal well-being (8, 9). To provide a concise overview of these core constructs and their operationalization in the context of adolescents and college students physical activity, Table 1 summarizes the definitions and example measurement items for each TPB component, along with the extended construct of behavior plan.

**Table 1:** Constructs of the theory of planned behavior (TPB) and example measurement items.

Construct	Definition	Example measurement items (for physical activity)
Attitude (ATT)	The degree to which a person has a favorable or unfavorable evaluation of the behavior.	“For me, engaging in at least 30 min of moderate-to-vigorous physical activity on most days of the week would be.”
Subjective norm (SN)	The perceived social pressure from significant others to perform or not perform the behavior.	“Most people who are important to me think I should engage in regular physical activity.” “My close friends and family expect me to be physically active.”
Perceived behavioral control (PBC)	The perceived ease or difficulty of performing the behavior, reflecting past experiences and anticipated obstacles.	“How much personal control do you have over engaging in regular physical activity?” “For me, being physically active regularly is.”
Behavioral intention (BI)	An indication of how much effort a person is willing to exert to perform the behavior.	“I intend to engage in physical activity at least 5 times per week over the next month.” “How likely is it that you will exercise regularly in the coming weeks?”
Behavior plan (BP)	A self-regulatory strategy specifying the precise when, where, and how of goal pursuit.	“I have already made concrete plans regarding exactly when and where I will exercise next week.” “I have a detailed schedule for my physical activities.”

ATT = Attitude; SN = Subjective Norm; PBC = Perceived Behavioral Control; BI = Behavioral Intention; BP = Behavior Plan. Definitions are based on the original TPB formulations Ajzen (12) and the implementation intention framework Gollwitzer (26). BP is not part of the original TPB but is included as an extended construct in the present meta-analysis.

The Theory of Planned Behavior (TPB) is a widely applied framework for understanding and guiding health-related behaviors, covering areas such as cancer screening, smoking cessation, and physical activity participation (10, 11). The model posits that intention is the most direct determinant of behavior, which is in turn influenced by three factors: attitude (an individual’s positive or negative evaluation of the behavior), subjective norm (SN; perceived social pressure to perform the behavior), and perceived behavioral control (PBC; perceived ease or difficulty of performing the behavior). While these factors typically influence behavior indirectly through intention, PBC can also directly influence behavior when it accurately reflects actual control (12). According to this theory, when young people form a more favorable attitude, feel stronger positive social pressure, and perceive greater control over physical activities, they are more likely to develop a stronger intention to participate.

TPB has been successfully applied to predict the physical activity of adolescents (13–15) and college students (16–18). However, despite its utility, a considerable proportion of variance in behavior remains unexplained. A meta-analysis of 72 TPB studies on physical activities showed that the model explained 45 and 27% of behavioral intention and behavioral variation, respectively (19). This limitation has led researchers to suggest extending TPB by incorporating additional predictive variables (19, 20). Consequently, recent studies have expanded the model with constructs such as self-identity (21), past behavior (22), social support (23), and key behavior plan (24, 25).

A behavior plan is a forward-looking self-regulation strategy that facilitates the translation of behavioral intentions into actual actions. While behavioral intention reflects an individual’s readiness and willingness to take action, a behavior plan can theoretically simulate the psychological connection between situational cues and specific behavioral responses, thereby increasing the likelihood of action initiation (26). Empirical research supports its mechanism of action; for instance, an intervention that includes behavior plan is more effective in increasing physical activity than interventions that target intention alone (27). This aligns with models such as the Health Action Process Approach (HAPA), which positions behavior plan as a key mediator variable between behavior intention and behavior (28). Behavior plan demonstrates significant effects on both the initiation and maintenance of behavior (29).

Meta-analysis is a powerful tool for integrating different research findings and resolving conflicting results. Previous meta-analysis studies have established the general applicability of TPB in explaining the intentions and behaviors of physical activities (11, 19). And it was pointed out that demographic factors such as age may have a moderating effect on the relationships within the TPB model (19, 30). Given that the adolescents and college students population encompasses distinct subgroups such as adolescents and college students, who may experience different social environments and developmental contexts, it is plausible that such demographic factors, alongside other study-level characteristics, may serve as key moderating variables in the extended TPB framework.

However, a significant gap remains in existing literature. Presently, there is no meta-analysis review that has specifically synthesized the TPB extended model for predicting physical activity participation exclusively in the adolescents and college student’s population, particularly models incorporating behavior plans. The application of rigorous meta-analysis structural equation modeling (MASEM) can significantly enhance the accuracy and interpretability of the TPB extended model in predicting adolescents and college student’s behavioral patterns. By synthesizing existing data in this manner, this study aims to provide high-quality, evidence-based guidance for the development of policies and intervention measures to promote physical activities among adolescents and college students based on the extended TPB as a theoretical foundation.

Therefore, the main objectives of this study include: (1) conducting a meta-analysis of the structure of the TPB extended model to examine the predictive effects of each component of the model on the participation of physical activities in the adolescents and college students population; (2) systematically examining potential moderating variables in the extended TPB relationship, including cultural background, physical activity measurement method, study design, and gender composition, in addition to the participant groups (adolescents and college students); (3) testing the mediating effect of the TPB extended model; (4) the direct impact of perceived behavioral control on behavior among adolescents and college students people.

## Methods

### Meta-analysis

Generally speaking, there are two main methods used to review published research: traditional qualitative methods and meta-analysis. Meta analysis is a statistical quantification approach that calculates the effect sizes of the reviewed studies (31), which is based on synthesizing conflicting findings from a large number of studies (32). Therefore, some scholars believe that meta-analysis is a more suitable method. Compared with traditional qualitative methods, meta-analysis adopts mathematical statistical methods to integrate existing research results (33), aiming to merge and summarize various research conclusions to elucidate the current state of knowledge and provide direction for future research.

### Literature search and research coding

The present study was conducted following established meta-analysis guidelines (34, 35). To identify relevant literature, a systematic search was conducted across multiple electronic databases including Web of Science, Scopus, ProQuest, Google Scholar, CNKI, and Wanfang. The search strategy utilized the following key terms and their combinations in “all fields”: “Extended TPB model” OR “TPB model,” AND “pa” OR “mvpa” OR “physical activity” OR “exercise behavior” OR “physical exercise.” After removing duplicate literature, the retrieved studies were screened according to pre-set inclusion criteria (36): (1) they must be empirical studies reporting correlation coefficients; (2) at least one of the above hypotheses is included in the study; (3) the definitions of antecedent variables and outcome variables must be consistent with the conceptual definitions in the previous text; (4) Participant characteristics. The target population for this review was adolescents and college students (approximately 10–24 years), encompassing a developmental continuum from middle adolescence to emerging adulthood. Studies were included if they explicitly described their participants as “adolescents,” “teenagers,” “middle/high school students”, or “college/university students.” Consistent with the WHO definition of adolescence (10–19 years) and the typical age range of Chinese college students (18–22 years), we adopted 10–24 years as the operational age range for this review. Although the United Nations defines “youth” as 15–24 years, a rigid cutoff of 15 years would have excluded a substantial body of relevant research on middle school students (typically aged 12–15 years). Therefore, we included studies if they met either of the following criteria: (a) the sample was explicitly identified as “adolescent,” “teenager,” or “middle/high school student”; or (b) the sample was identified as “college student” or “undergraduate.” For studies with mixed-age samples spanning adolescence and adulthood, we included them only if separate data for the younger subgroup could be extracted or if the mean age did not exceed 30 years. Studies exclusively targeting graduate students (postgraduates), older adults, or clinical populations outside this age range were excluded. (5) English literature was limited to SCI and SSCI indexed journals, while Chinese literature was required to be published in Chinese core journals or higher.

After this screening process, 28 studies comprising 31 independent samples were retained for the analysis. The detailed process of study identification and screening is summarized in Figure 1.

**Figure 1:**
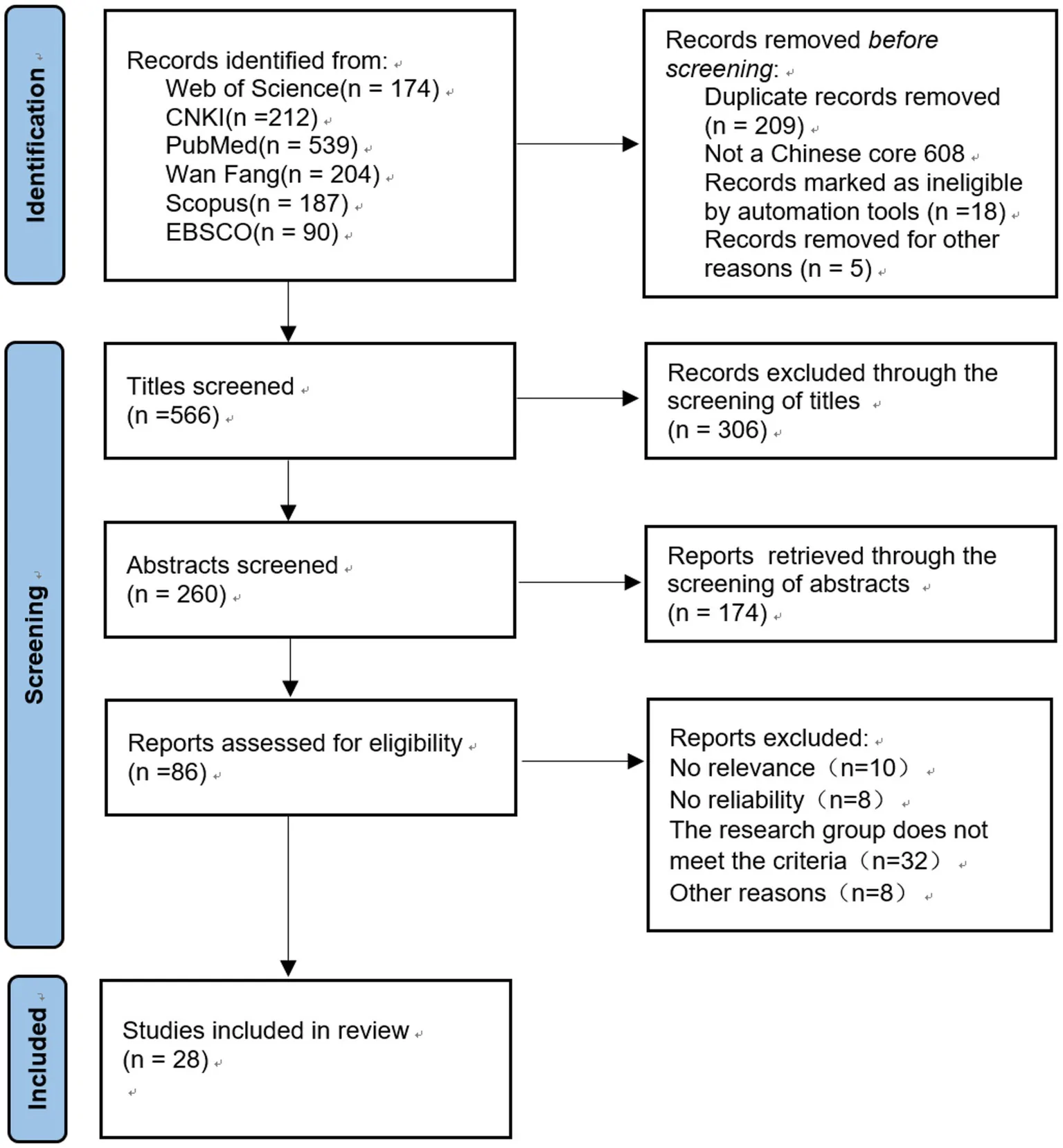
PRISMA flowchart for study selection.

### Coding of behavior plan

In this meta-analysis, “behavior plan” was defined as any self-regulatory strategy that clearly specifies when, where, and how an individual intends to engage in physical activity, consistent with the conceptualization of implementation intentions and behavior plan (26). The study is considered to have measured behavior plan if it includes any scales assessing behavior plan, coping plans, implementation intentions, or related constructs (such as “I have made a detailed exercise schedule”). Based on this definition, the specific instruments used in each study (such as Planning Scale, Implementation Intention Scale, and Self-Reporting Habits Index) were coded from the original literature. The detailed coding results are presented in Supplementary Table S1 (Supplementary material S1; column “BP measure detail”).

### Quality assessment

The methodological quality of the included studies was assessed independently by two reviewers (YY and YQ) using the Newcastle-Ottawa Scale adapted for cross-sectional studies (NOS-xs) (37). The NOS-xs scale evaluates studies from three domains: selectivity (4 items, 1 star each), comparability (2 items, 1 star each), and outcome (3 items, 1 star each), with a maximum total of 9 stars. The risk of research bias is classified as low rise (7–9 stars), moderate risk (4–6 stars), and high risk (0–3 stars) based on scoring. Any disagreements were resolved through discussion or consultation with a third reviewer (GL).

## Statistical methods

All meta-analysis computations were completed using Comprehensive Meta-Analysis (CMA) software version 3.0. The hypothesized structural model was tested using Amos 24.0.

The correlation coefficient (r) was selected as the effect size metric due to its interpretability and widespread application in social science meta-analyses (34). When other statistical indicators (such as *χ*^2^, t, F) were used in the study, they were converted to *r*-values using standard formulas (36). Because many primary studies did not report full reliability coefficients (Cronbach’s α) for all constructs, measurement-error correction was not applied at the individual effect-size level. The observed correlation coefficients were directly pooled using CMA 3.0.

The correlation coefficients were then synthesized using a random-effects model, which can handle heterogeneity beyond sampling error range (38). The effect size was weighted by the inverse of its variance to give higher weight to studies with larger sample sizes. For each pooled effect size, we reported the point estimates along with its 95% confidence intervals. To assess potential publication bias, we employed multiple methods. First, the classic fail-safe N was calculated. Second, funnel plots were visually inspected for data asymmetry. Third, Egger’s regression intercept test was conducted to statistically examine small sample effects, with *p* < 0.10 as the threshold for potential publication bias (39). These analyses were performed for all pairwise relationships using CMA 3.0.

In addition to the pairwise meta-analysis, we conducted two-stage meta-analytic structural equation modeling (MASEM) to examine the relationships among the most frequently reported behavior antecedents and outcomes. In Stage 1, random-effects pairwise meta-analyses were conducted to obtain a pooled correlation matrix using CMA 3.0. In Stage 2, this pooled correlation matrix was analyzed using conventional SEM in Amos 24.0, with the harmonic mean of the total sample sizes across all correlations used as the effective sample size (40). The construct was included in the MASEM analysis only if correlation data between that construct and all other constructs in the model was available in the literature (41). We required a minimum of three studies per correlation in the input matrix for inclusion of a construct, ensuring the stability of the estimated values (38). For the behavior plan construct in subgroup analyses, this threshold could not always be met due to limited primary studies (e.g., *k* = 1–2 for some BP correlations in the adolescent subgroup); these estimates were retained to preserve model completeness but are explicitly flagged as exploratory. Further subgroup-specific sensitivity analyses (e.g., leave-one-out) were not feasible given the small number of contributing studies (*k* ≤ 2); thus, these subgroup paths are presented for hypothesis-generating purposes only. Because the pooled correlation matrix contained observed rather than disattenuated correlations, the error variances of the observed variables were constrained based on their unreliability (1 − α) in the SEM to account for measurement error (42). This two-stage approach differs from one-stage MASEM frameworks (43) that simultaneously model study-level correlation matrices and sample sizes within a unified random-effects model.

This review was conducted and reported in accordance with the Preferred Reporting Items for Systematic Reviews and Meta-Analyses (PRISMA) guidelines. The protocol was registered with the International Platform of Registered Systematic Review and Meta-analysis Protocols (INPLASY) on 8 March 2024 under the registration number INPLASY202430027, and is available at: https://doi.org/10.37766/inplasy2024.3.0027.

## Results

### Study characteristics

A total of 28 articles comprising 31 independent samples met the inclusion criteria. Among these, three articles provided separate data for different subgroups: Luszczynska et al. (44) reported two independent samples from China and Poland; Feng et al. (25) reported correlation data for male and female college students separately; and Blanchard et al. (16) reported data for Black and White students, respectively. The remaining 25 articles each contributed one independent sample. Consistent with our operational definition (adolescents and college students, approximately 10–24 years). Of the 31 samples, 11 focused on adolescents and 20 targeted college students. Studies were conducted in China, the United States, Canada, Spain, and Poland, with sample sizes ranging from 134 to 2,302, totaling 19,652 unique participants.

Regarding behavior plan, 10 out of the 31 independent samples (32.3%) measured behavior plan, coping plans, or implementation intentions. Studies measuring habit, mental toughness, executive function, barriers, motivation, or other constructs conceptually distinct from prospective plan were excluded from the BP synthesis to ensure construct validity (see Table S1).

### Quality assessment results

The quality assessment results of the 28 included studies are presented in Supplementary Table S2 (Supplementary material S2). The total NOS-xs scores ranged from 5 to 8, with a mean of 6.96 (Standard Deviation = 0.793). Among the 28 studies, 21 (75%) were rated as low risk of bias (≥7 stars), and 7 (25%) as moderate risk of bias (4–6 stars). No study was classified as high risk. The most common methodological weaknesses were inadequate control of confounding factors and lack of justification for sample size. Sensitivity analysis excluding the seven studies with moderate risk of bias yielded consistent results with the main analyses, indicating the robustness of the study findings.

### Pairwise meta-analysis

We first examined the bivariate relationships among all TPB constructs. These zero-order correlations provide the foundation for the subsequent structural equation model and reveal the overall strength and direction of associations between each pair of constructs. The present meta-analysis synthesized 263 effect sizes from 31 independent samples, encompassing a total of 133,732 participants (cumulative across pairwise comparisons; unique participant total = 19,652). The analysis results of all pairwise relationships are presented in Table 2.

**Table 2:** Analysis of bivariate correlation.

Pairwise relationships	K	N	Weighted average correlation		95% Cl		I squared	Fail-safe N	Q (df)
			Correlation	SE	Lower limit	Upper limit			
ATT to B	21	9,499	0.263	0.030	0.206	0.317	88.704	3,718	162.648 (20)
ATT to BI	23	10,196	0.456	0.034	0.397	0.511	92.412	3,130	289.947 (22)
ATT to BP	5	2090	0.258	0.105	0.053	0.442	95.555	148	89.994 (4)
ATT to PBC	22	9,768	0.425	0.055	0.327	0.514	96.893	773	675. 966 (21)
ATT to SN	23	10,196	0.298	0.056	0.192	0.397	96.936	5,132	717.955 (22)
BI to B	28	17,499	0.425	0.039	0.355	0.489	96.550	13,758	782.665 (27)
BI to BP	8	4,336	0.434	0.073	0.301	0.550	96.053	1796	177.349 (7)
PBC to B	22	12,465	0.303	0.029	0.248	0.355	90.210	5,593	214.497 (21)
PBC to BI	24	13,162	0.528	0.037	0.467	0.584	95.316	14,097	491.031 (23)
PBC to BP	6	3,182	0.468	0.134	0.220	0.660	98.380	1,174	308.634 (5)
BP to B	8	4,336	0.342	0.053	0.244	0.434	91.937	1,063	86.820 (7)
SN to B	21	10,345	0.188	0.033	0.124	0.250	90.440	1738	209.215 (20)
SN to BI	24	12,498	0.410	0.044	0.331	0.484	96.114	3,207	591.897 (23)
SN to BP	5	2090	0.285	0.073	0.145	0.415	90.938	208	44.139 (4)
SN to PBC	23	12,070	0.318	0.052	0.221	0.409	96.937	6,235	718.219 (22)

K represents the number of studies or independent samples; N is the total number of samples; *r* is the average effect value; I squared is a homogeneity test; Failsafe *N* represents publishing bias.

The meta-analytic correlations represent average associations across highly heterogeneous studies (*I*^2^ often >90%; see Table 2) and indicate that all hypothesized relationships within the extended TPB model were statistically significant on average. Attitude was on average significantly correlated with all other constructs: subjective norms (SN, *r* = 0.298), perceived behavioral control (PBC, *r* = 0.425), intention (*r* = 0.456), behavior plan (*r* = 0.258), and behavior (*r* = 0.263). Similarly, subjective norms were significantly correlated with PBC (*r* = 0.318), intention (*r* = 0.410), behavior plan (*r* = 0.285), and behavior (*r* = 0.188). Notably, the relationship between subjective norms and intention was among the strongest for this construct. Perceived behavioral control also showed significant, medium-to-large correlations with intention (*r* = 0.528), behavior plan (*r* = 0.468), and behavior (*r* = 0.303).

As predicted, intention was strongly correlated with both behavior (*r* = 0.425) and behavior plan (*r* = 0.434). Furthermore, a significant positive correlation was found between behavior plan and behavior (*r* = 0.342). According to Cohen’s (45) conventions, the observed average effects ranged from medium to large. However, substantial heterogeneity was observed across studies, with *I*^2^ values often exceeding 90% (see Table 2). Consequently, these pooled correlation coefficients reflect broad averages across diverse populations, instruments, cultures, and study designs rather than precise or generalizable estimates of a single true effect. Any interpretation of their magnitude or practical significance should be tempered by this considerable cross-study variability. Finally, the classic fail-safe N was calculated to assess publication bias. For all pairwise relationships, the fail-safe N exceeded 100, suggesting that the results are robust against the “file drawer problem” (46).

### Publication bias

In addition to the classic fail-safe *N*, we also assessed the publication bias of all pairwise relationships through funnel plots and Egger’s regression tests. The results are summarized in Supplementary Table S3 (Supplementary material S3). Most of the associated Egger’s tests showed no statistical significance (all *p*-values>0.05), and the funnel plots presented a symmetrical distribution, indicating no evidence of publication bias. The sole exception was the ATT → B relationship, for which Egger’s test was significant (intercept = −4.321, *p* = 0.027), suggesting the potential for small sample study effects. The visual inspection of the funnel plot for ATT → B revealed slight asymmetry, with a relative absence of studies in the left lower quadrant. However, the fail-safe N for all pairwise associations exceeded 100, and the overall meta-analytic conclusions appear to be relatively robust, although the ATT → B estimate should be interpreted with caution given the potential for small-study effects.

### Moderator analysis

To explore the substantial heterogeneity observed in all pairwise relationships (with *I*^2^ values often exceeding 90%), we conducted moderator analyses on five study-level characteristics: group type (adolescents vs. college students), cultural background (Eastern vs. Western), physical activity measurement method (objective measurement vs. self-report), study design (prospective vs. cross-sectional), and percentage of female participants (meta-regression). The detailed results are presented in Table 3.

**Table 3:** Moderator analyses for key TPB relationships.

Path	Moderator	Subgroup/variable	k	r (or β for meta-regression)	95% CI	Test of difference Q(p)
ATT to B	Group type	Adolescents	6	0.191	[0.090, 0.289]	3.167 (0.075)
		College students	15	0.295	[0.237, 0.351]	
	Culture	Eastern	16	0.264	[0.197, 0.3328]	0.004 (0.948)
		Western	5	0.260	[0.159, 0.356]	
	PA measure type	Objective	2	0.125	[0.020, 0.227]	6.787 (0.034)
		Self-report	18	0.280	[0.222, 0.336]	
	Study design	Prospective	7	0.230	[0.120, 0.335]	0.550 (0.458)
		Cross-sectional	14	0.278	[0.212, 0.340]	
	Female (%)	Meta-regression	21	0.091	[−0.250, 0.433]	*P* = 0.601
ATT to BI	Group type	Adolescents	6	0.445	[0.305, 0.566]	0.040 (0.841)
		College students	17	0.460	[0.393, 0.522]	
	Culture	Eastern	18	0.450	[0.378, 0.516]	0.187 (0.665)
		Western	5	0.473	[0.392, 0.546]	
	PA measure type	Objective (accelerometer)	2	0.305	[0.252, 0.356]	18.755 (0.001)
		Self-report	20	0.470	[0.406, 0.529]	
	Study design	Prospective	8	0.514	[0.427, 0.592]	2.493 (0.114)
		Cross-sectional	15	0.424	[0.347, 0.495]	
	Female (%)	Meta-regression	23	0.157	[−0.272, 0.585]	*p* = 0.474
ATT to BP	Group type	Adolescents	1	0.048	[−0.021, 0.116]	4.867 (0.027)
		College students	4	0.309	[0.088 0.502]	
	Culture	Eastern	5	0.258	[0.053, 0.442]	–
		Western	0	–	–	
	PA measure type	Objective	1	0.048	[−0.021, 0.116]	4.867 (0.027)
		Self-report	4	0.309	[0.088, 0.502]	
	Study design	Prospective	0	–	–	–
		Cross-sectional	5	0.258	[0.053, 0.442]	
	Female (%)	Meta-regression	5	0.214	[−0.504, 0.934]	*p* = 0.558
ATT to PBC	Group type	Adolescents	5	0.422	[0.255, 0.565]	0.002 (0.968)
		College students	17	0.426	[0.301, 0.537]	
	Culture	Eastern	18	0.451	[0.344, 0.56]	2.319 (0.128)
		Western	4	0.298	[0.114, 0.461]	
	PA measure type	Objective	2	0.328	[0.276, 0.378]	3.068 (0.080)
		Self-report	20	0.434	[0.326, 0.531]	
	Study design	Prospective	8	0.461	[0.181, 0.672]	0.174 (0.677)
		Cross-sectional	14	0.404	[0.323, 0.479]	
	Female (%)	Meta-regression	22	−0.030	[−0.700, 0.639]	*p* = 0.929
ATT to SN	Group type	Adolescents	6	0.358	[0.206, 0.493]	0.660 (0.417)
		College students	17	0.276	[0.137, 0.404]	
	Culture	Eastern	18	0.291	[0.162, 0.410]	0.181 (0.670)
		Western	5	0.326	[0.218, 0.426]	
	PA measure type	Objective	2	0.154	[0.097, 0.209]	40.040 (0.000)
		Self-report	20	0.302	[0.182, 0.414]	
	Study design	Prospective	8	0.415	[0.261, 0.548]	3.232 (0.072)
		Cross-sectional	15	0.234	[0.101, 0.358]	
	Female (%)	Meta-regression	23	0.250	[−0.972, 0.470]	*p* = 0.500
BI to B	Group type	Adolescents	11	0.351	[0.271, 0.426]	3.611 (0.057)
		College students	17	0.471	[0.372, 0.560]	
	Culture	Eastern	22	0.408	[0.328, 0.483]	0.927 (0.336)
		Western	6	0.484	[0.345, 0.602]	
	PA measure type	Objective	2	0.325	[0.273, 0.376]	14.023 (0.001)
		Self-report	25	0.430	[0.354, 0.500]	
	Study design	Prospective	9	0.423	[0.286, 0.544]	0.000(0.983)
		Cross-sectional	19	0.425	[0.341, 0.502]	
	Female (%)	Meta-regression	28	0.053	[−0.478, 0.584]	*p* = 0.844
BI to BP	Group type	Adolescents	4	0.333	[0.116, 0.519]	3.349 (0.067)
		College students	4	0.528	[0.436, 0.609]	
	Culture	Eastern	7	0.444	[0.290, 0.576]	1.016(0.313)
		Western	1	0.360	[0.289, 0.427]	
	PA measure type	Objective	1	0.088	[0.020, 0.156]	43.687 (0.000)
		Self-report	7	0.476	[0.389, 0.555]	
	Study design	Prospective	2	0.337	[0.285, 0.387]	1.926 (0.165)
		Cross-sectional	6	0.466	[0.287, 0.613]	
	Female (%)	Meta-regression	9	−0.023	[−0.664, 0.618]	*p* = 0.944
PBC to B	Group type	Adolescents	7	0.268	[0.138, 0.390]	0.581 (0.446)
		College students	15	0.319	[0.279, 0.358]	
	Culture	Eastern	18	0.314	[0.253, 0.373]	3.355 (0.067)
		Western	4	0.228	[0.157, 0.297]	
	PA measure type	Objective	2	0.189	[0.122, 0.255]	7.759 (0.005)
		Self-report	20	0.313	[0.256, 0.369]	
	Study design	Prospective	7	0.288	[0.196, 0.374]	0.135 (0.714)
		Cross-sectional	15	0.308	[0.241, 0.372]	
	Female (%)	Meta-regression	22	0.007	[−0.339, 0.354]	*p* = 0.967
PBC to BI	Group type	Adolescents	7	0.542	[0.479, 0.600]	0.128 (0.721)
		College students	17	0.522	[0.426, 0.607]	
	Culture	Eastern	20	0.536	[0.470, 0.595]	0.266 (0.606)
		Western	4	0.488	[0.293, 0.644]	
	PA measure type	Objective	2	0.548	[0.507, 0.587]	0.339 (0.561)
		Self-report	22	0.526	[0.458, 0.588]	
	Study design	Prospective	8	0.566	[0.454, 0.659]	0.727 (0.394)
		Cross-sectional	16	0.510	[0.431, 0.580]	
	Female (%)	Meta-regression	24	−0.049	[−0.546, 0.449]	*p* = 0.847
PBC to BP	Group type	Adolescents	2	0.401	[−0.309, 0.824]	0.100(0.752)
		College students	4	0.500	[0.342, 0.630]	
	Culture	Eastern	6	0.468	[0.220, 0.660]	–
		Western	0	–	–	
	PA measure type	Objective	1	0.045	[−0.024, 0.113]	29.581(0.000)
		Self-report	5	0.539	[0.392, 0.659]	
	Study design	Prospective	0	–	–	–
		Cross-sectional	6	0.468	[0.220, 0.660]	
	Female (%)	Meta-regression	6	0.044	[−1.006, 1.095]	*p* = 0.934
BP to B	Group type	Adolescents	4	0.348	[0.190, 0.518]	0.279 (0.597)
		College students	4	0.318	[0.267, 0.367]	
	Culture	Eastern	7	0.332	[0.219, 0.437]	1.465 (0.226)
		Western	1	0.410	[0.343, 0.473]	
	PA measure type	Objective	1	0.109	[0.041, 0.176]	31.403 (0.000)
		Self-report	7	0.377	[0.313, 0.439]	
	Study design	Prospective	2	0.472	[0.346, 0.581]	4.878 (0.027)
		Cross-sectional	6	0.294	[0.194, 0.389]	
	Female (%)	Meta-regression	8	0.102	[−0.349, 0.548]	*p* = 0.656
SN to B	Group type	Adolescents	7	0.170	[0.093, 0.245]	0.185 (0.667)
		College students	14	0.197	[0.098, 0.293]	
	Culture	Eastern	16	0.167	[0.092, 0.239]	3.129 (0.077)
		Western	5	0.266	[0.183, 0.346]	
	PA measure type	Objective	2	0.069	[0.012, 0.126]	37.056 (0.000)
		Self-report	18	0.189	[0.121, 0.256]	
	Study design	Prospective	7	0.228	[0.144, 0.308]	1.008 (0.315)
		Cross-sectional	14	0.168	[0.084, 0.249]	
	Female (%)	Meta-regression	21	0.007	[−0.369, 0.383]	*p* = 0.970
SN to BI	Group type	Adolescents	7	0.419	[0.278, 0.542]	0.020 (0.888)
		College students	17	0.407	[0.304, 0.500]	
	Culture	Eastern	19	0.416	[0.323, 0.501]	0.123 (0.726)
		Western	5	0.391	[0.276, 0.494]	
	PA measure type	Objective	2	0.201	[0.145, 0.255]	56.416 (0.000)
		Self-report	21	0.422	[0.340, 0.498]	
	Study design	Prospective	8	0.430	[0.315 0.533]	0.163 (0.687)
		Cross-sectional	16	0.400	[0.297, 0.494]	
	Female (%)	Meta-regression	24	0.011	[−0.547, 0.570]	*p* = 0.968
SN to BP	Group type	Adolescents	1	0.173	[0.106, 0.239]	2.345 (0.126)
		College students	4	0.314	[0.144, 0.466]	
	Culture	Eastern	5	0.285	[0.145, 0.415]	–
		Western	0	–	–	
	PA measure type	Objective	1	0.173	[0.106, 0.239]	2.345 (0.126)
		Self-report	4	0.314	[0.144, 0.466]	
	Study design	Prospective	0	-	-	–
		Cross-sectional	5	0.285	[0.145, 0.415]	
	Female (%)	Meta-regression	5	0.104	[−0.414, 0.622]	*p* = 0.694
SN to PBC	Group type	Adolescents	6	0.402	[0.299, 0.495]	1.826 (0.177)
		College students	17	0.287	[0.146, 0.417]	
	Culture	Eastern	19	0.312	[0.205, 0.412]	0.066 (0.798)
		Western	4	0.351	[0.051, 0.593]	
	PA measure type	Objective	2	0.303	[0.250, 0.355]	0.077 (0.782)
		Self-report	21	0.320	[0.212, 0.420]	
	Study design	Prospective	8	0.435	[0.272, 0.575]	3.229 (0.072)
		Cross-sectional	15	0.253	[0.132, 0.367]	
	Female (%)	Meta-regression	23	0.185	[−0.439, 0.809]	*p* = 0.561

*k* = number of independent samples; *r* = pooled correlation coefficient; 95% CI = 95% confidence interval.

For the meta-regression of Female (%), the coefficient *β* is reported instead of *r*.

Effect sizes for female (%) are presented as *β* with 95% CI.

Group type: adolescents vs. college students.

Culture: Eastern (China) vs. Western (USA, Canada, Spain, Poland).

PA measure type: objective (accelerometer) vs. self-report (all questionnaires).

Study design: Prospective (longitudinal follow-up) vs. cross-sectional.

Female (%): meta-regression coefficient representing the change in effect size per 1 unit increase in the proportion of female participants. Because the predictor was entered as a proportion (0–1), a 1-unit increase corresponds to a 100 percentage point increase (e.g., from 0 to 100% female).

Subgroups containing fewer than three studies (*k* < 3) are presented for descriptive purposes only; statistical inference is not warranted for these comparisons.

The sum of subgroup k may be one less than the overall k for a given path when a study did not report or used a mixed physical activity measurement method. Such studies were excluded from the PA measure type moderator analysis.

Overall, most moderator variables fail to systematically explain the heterogeneity. For example, in the ATT → B relationship, no significant difference was found between Eastern and Western cultures (Q_between_ = 0.004, *p* = 0.948), or prospective and cross-sectional designs (Q_between_ = 0.550, *p* = 0.458). The meta-regression analysis of female percentage also showed no significant difference (*β* = 0.091, *p* = 0.601). Similar patterns were observed across most other paths. Coefficients represent the change in effect size per 1 unit (i.e., 100 percentage point) increase in the proportion of female participants.

However, several significant effects were identified. For instance, the relationship between attitude and behavioral intention was significantly moderated by the physical activity measurement method, with self-report studies (*r* = 0.470) yielding significantly greater effects than objective studies (*r* = 0.305; Q between = 18.755, *p* = 0.001). These findings suggest that while the TPB relationships are generally robust, specific study characteristics may influence the strength of certain paths. For paths where some subgroups contained fewer than three studies (e.g., ATT → BP, SN → BP, PBC → BP), the results are reported descriptively due to insufficient sample sizes for reliable statistical inference. This caveat applies particularly to the behavior plan paths in the adolescent subgroup model (Figure 2), where the underlying correlations were based on one or two studies. Accordingly, the BP-related parameter estimates for adolescents are presented for exploratory completeness only and should not be used to draw firm inferences about the role of plan in this age group.

**Figure 2:**
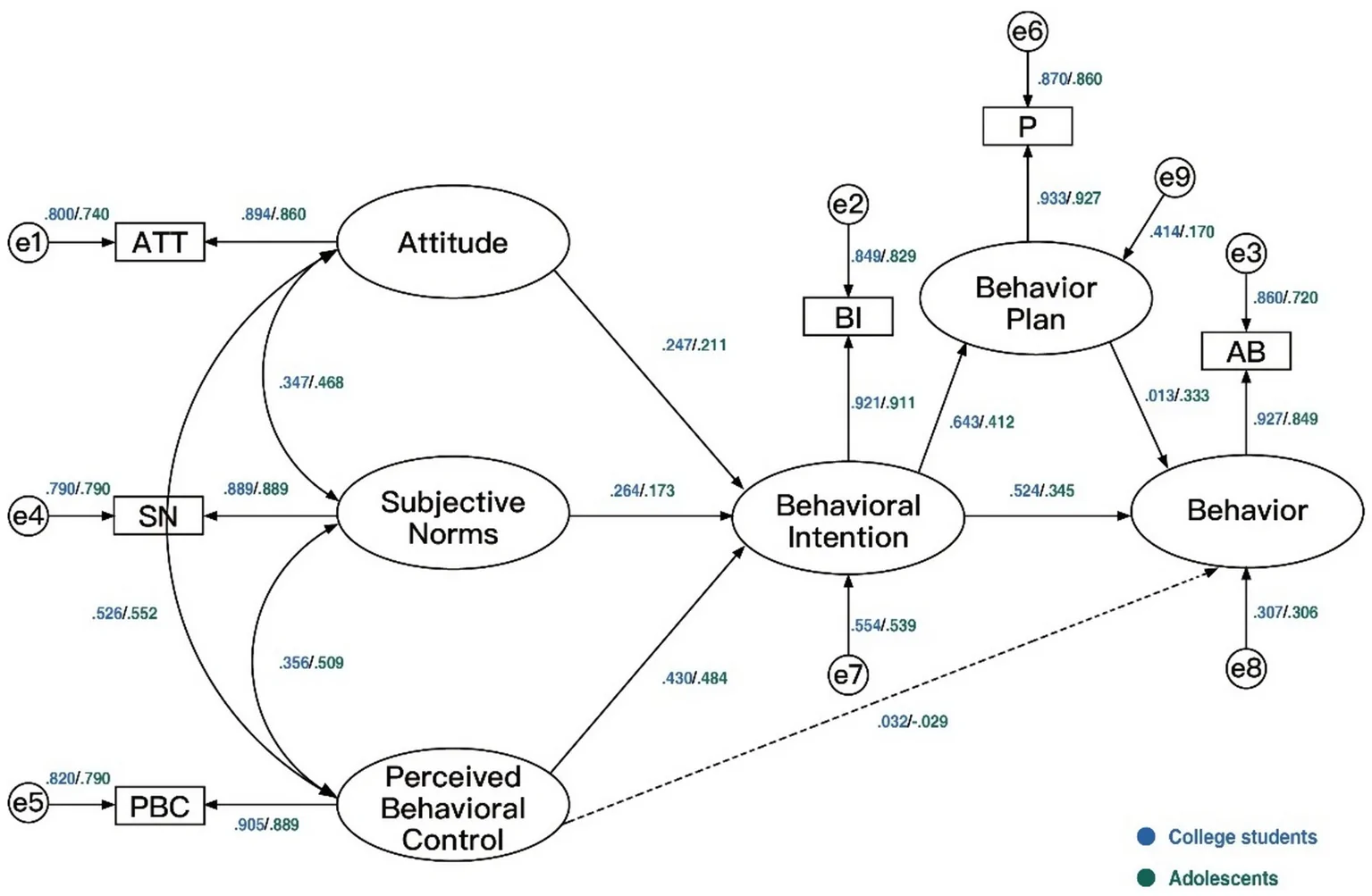
Meta-analytic structural equation modeling with adolescents and college students samples. Path coefficients are standardized *β* values. Solid lines indicate significant paths (*p* < 0.05); dashed lines indicate non-significant paths (*p* > 0.05).

### Meta-analytic structural equation Modeling

To test all hypothesized pathways simultaneously while accounting for the pooled covariance structure, we next conducted meta-analytic structural equation modeling (MASEM). This approach integrates the pairwise meta-analytic correlations into a unified path model, allowing us to estimate direct, indirect, and total effects. Table 4 shows the correlation matrix for conducting MASEM analysis, and Figure 3 presents the results of MASEM. The overall model (Figure 3) revealed mixed fit indices, with *χ*^2^ (3) = 452.911; *p* < 0.001, goodness-of-fit index (GFI) = 0.975, comparative fit index (CFI) = 0.944, normed fit index (NFI) = 0.943, adjusted goodness-of-fit index (AGFI) = 0.894, and root mean square residual (RMR) = 0.041. While CFI approached the conventional threshold of 0.95, AGFI fell slightly below 0.90, indicating borderline fit. These fit indices should be interpreted with caution given the two-stage estimation procedure and the use of harmonic mean sample sizes. All hypothesized paths were significant (*p* < 0.001) except for the direct path from perceived behavioral control to behavior (*β* = 0.006, *p* = 0.791), with the extended TPB model accounting for 54.7% of the variance in intention, 29.2% of the variance in behavior and 28.9% of the variance in behavior plan. Specifically, Perceived Behavioral Control (PBC; *β* = 0.436) exhibited the strongest statistical association with behavioral intention (BI), followed by Subjective Norms (SN; *β* = 0.242) and Attitude (ATT; *β* = 0.241). Moreover, BI had a strong impact on behavior plan (BP; *β* = 0.538) and a positive influence on behavior (B; *β* = 0.418), whereas BP also had a positive influence on B (*β* = 0.180). It is worth noting that, Perceived Behavioral Control did not have a positive influence on behavior (*β* = 0.006).

**Table 4:** Pooled correlation matrix used for MASEM analysis.

Constructs	ATT	SN	PBC	BI	BP	B
ATT	0.780					
SN	0.298	0.794				
PBC	0.425	0.318	0.808			
BI	0.456	0.410	0.528	0.847		
BP	0.258	0.285	0.468	0.434	0.869	
B	0.263	0.188	0.303	0.425	0.342	0.807

Values below the diagonal are the pooled correlation coefficients (*r*). Diagonal values denote Cronbach’s α. Harmonic mean of *N* = 5749.

**Figure 3:**
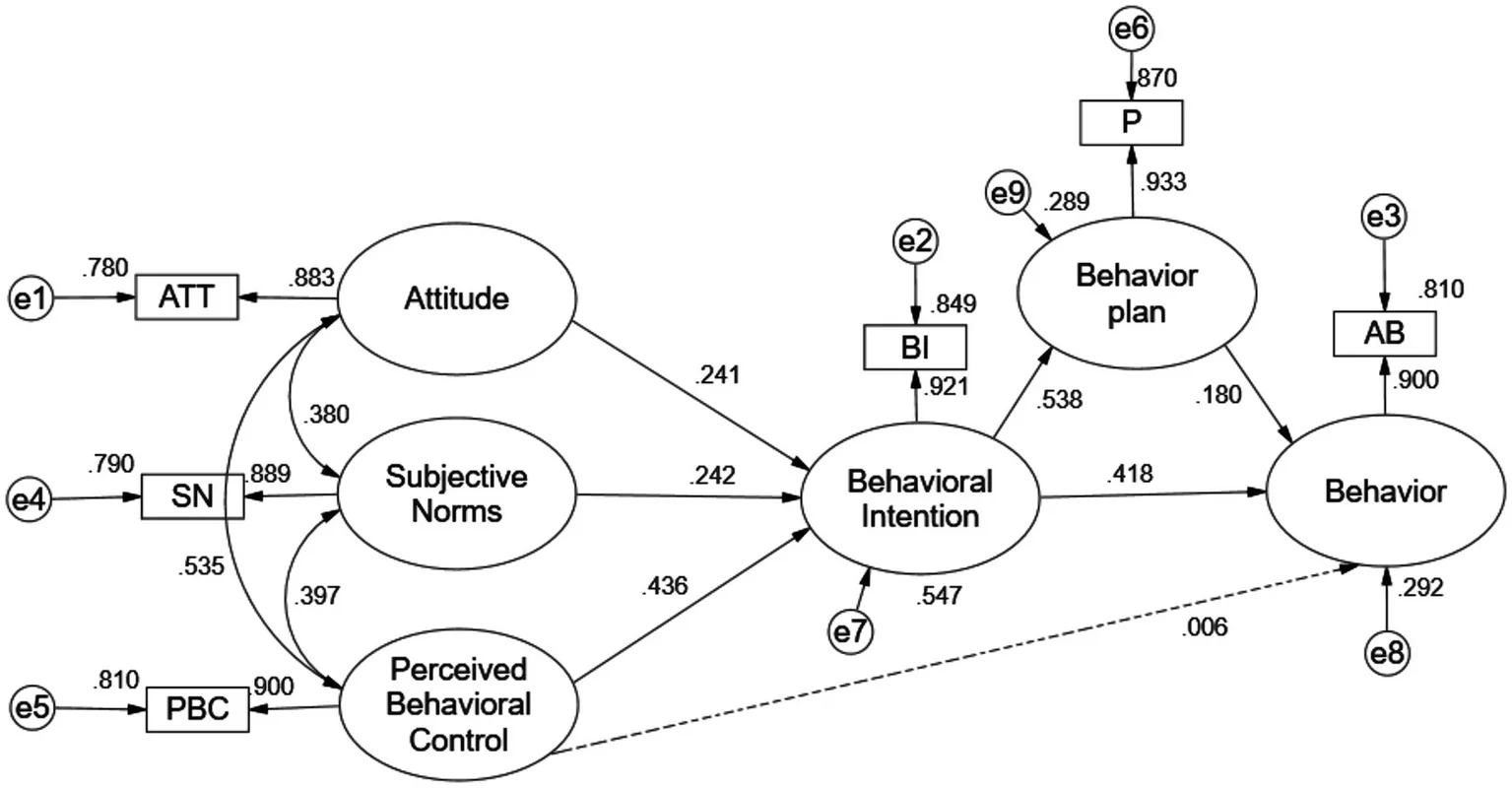
Meta-analytic structural equation modeling with the total sample. Path coefficients are standardized *β* values. Solid lines indicate significant paths (*p* < 0.05); the dashed line indicates the non-significant path (*p* > 0.05).

### Mediation effect test

The hypothesized statistical intermediary effects in the extended TPB model were tested using bootstrapping with 5,000 resamples. As summarized in Table 5, the mediation analysis results provided comprehensive support for the model.

**Table 5:** Results of the extended TPB model.

Variable path	Point estimation value	Coefficient product		Bootstrapping			
				Bias-corrected		Percentile	
				95% CI		95% CI	
		SE	Z	Lower	Upper	Lower	Upper
ATT->BI->B	0.100	0.009	11.111	0.084	0.119	0.083	0.118
SN->BI->B	0.101	0.008	12.625	0.087	0.117	0.086	0.117
PBC->BI->B	0.182	0.012	15.167	0.159	0.207	0.159	0.206
BI->BP->B	0.097	0.010	9.700	0.077	0.116	0.078	0.117
ATT->BI->BP ->B	0.023	0.003	7.667	0.018	0.029	0.018	0.029
SN->BI->BP ->B	0.023	0.003	7.667	0.018	0.029	0.018	0.029
PBC->BI->BP ->B	0.042	0.005	8.400	0.034	0.052	0.034	0.052

Bootstrap 5000 Monte Carlo was used.

The results indicated that behavioral intention accounted for a significant proportion of the shared variance between the three core TPB antecedent variables and behavior. Similarly, behavior plan explained a significant portion of the covariance between behavioral intention and behavior. Because most primary studies were cross-sectional, these patterns reflect statistical dependencies rather than causal mechanisms between the three core TPB antecedent variables and behavior. The strongest mediation effect was observed in the path of perceived behavioral control through behavioral intention [Effect value = 0.182, 95% CI (0.159, 0.207)], followed by subjective norms (Effect value = 0.101, 95% CI [0.087, 0.117]) and attitude (Effect value = 0.100, 95% CI [0.084, 0.119]). Additionally, behavior plan significantly mediated the relationship between behavioral intention and behavior (Effect value = 0.097, 95% CI [0.077, 0.116]). The analysis further supported three significant sequential mediation paths. Specifically, the chain from subjective norms to behavior intention to plan to behavior (Effect value = 0.023, 95% CI [0.018, 0.029]), the chain from PBC to behavior intention to plan to behavior (Effect value = 0.042, 95% CI [0.034, 0.052]), and the chain from attitude to behavior intention to plan to behavior (Effect value = 0.023, 95% CI [0.018, 0.029]) were all statistically significant, accounting for 4.0, 7.4, and 4.0% of the total effect, respectively.

### Group-specific MASEM results

To elucidate the group differences identified in the moderator analysis, we conducted separate MASEMs for the adolescents and college student’s samples, using the correlation matrices presented in Tables 6 and 7, respectively.

**Table 6:** Pooled correlation matrix for adolescents sample.

Constructs	ATT	SN	PBC	BI	BP	B
ATT	0.741	3204(6)	2776(5)	3204(6)	817(1)	3204(6)
SN	0.358	0.790	5078(6)	5506(7)	817(1)	5506(7)
PBC	0.422	0.402	0.787	6170(7)	1909(2)	6170(7)
BI	0.445	0.419	0.542	0.831	3063(4)	9325(11)
BP	0.048	0.173	0.401	0.333	0.864	3063(4)
B	0.191	0.170	0.268	0.351	0.365	0.725

Values below the diagonal are the pooled correlation coefficients (*r*). Values above the diagonal present the total sample sizes and number of studies in parentheses. Values denote Cronbach α. Harmonic mean of *N* = 2536. The correlations involving behavior plan (BP) in the adolescent subsample were derived from a limited number of studies: ATT↔BP and SN↔BP were each based on *k* = 1 study, and PBC↔BP on *k* = 2 studies. These values were included to enable model estimation in an exploratory capacity; they do not meet the *k* ≥ 3 threshold applied elsewhere, and the resulting BP-related path coefficients in the adolescent SEM should not be interpreted as stable estimates.

**Table 7:** Pooled correlation matrix for college student’s sample.

Constructs	ATT	SN	PBC	BI	BP	B
ATT	0.803	6992(17)	6992(17)	6992(17)	1273(4)	6295(15)
SN	0.276	0.791	6992(17)	6992(17)	1273(4)	4839(14)
PBC	0.426	0.287	0.823	6992(17)	1273(4)	6295(15)
BI	0.460	0.407	0.522	0.851	1273(4)	8174(17)
BP	0.309	0.314	0.500	0.528	0.873	1273(4)
B	0.295	0.197	0.319	0.471	0.318	0.859

Values below the diagonal are the pooled correlation coefficients (*r*). Values above the diagonal present the total sample sizes and number of studies in parentheses. Values denote Cronbach *α*. Harmonic mean of *N* = 2761.

Both group-specific models demonstrated mixed to borderline fit to the data. The model for adolescents exhibited borderline fit indices (CFI = 0.920, AGFI = 0.858), with several indicators falling below conventional thresholds. For college students, the model fit was acceptable (CFI = 0.955, AGFI = 0.905), though similar interpretive caveats apply. Detailed fit statistics were as follows: adolescents—*χ*^2^(6) = 278.767, *p* < 0.001, GFI = 0.966, NFI = 0.919, RMR = 0.048; college students—*χ*^2^(7) = 192.588, *p* < 0.001, GFI = 0.977, NFI = 0.954, RMR = 0.035. It should be noted that the BP-related paths in the adolescent model (ATT → BP, SN → BP, PBC → BP, BP → B) rely on correlations derived from one to two studies (see Table 6). Consequently, these specific path estimates should be treated as exploratory rather than stable model parameters.

As illustrated in Figure 2, all hypothesized paths were significant (*p* < 0.001) except for the direct path from perceived behavioral control to behavior. The model accounted for 53.9% of the variance in intention and 30.6% in behavior. For adolescents, perceived behavioral control (PBC; *β* = 0.484) was the strongest predictor of behavioral intention (BI), followed by attitude (ATT; *β* = 0.211) and subjective norm (SN; *β* = 0.173). BI significantly predicted both behavior plan (BP; *β* = 0.412) and behavior (B; *β* = 0.345), and BP, in turn, was a significant predictor of B (*β* = 0.333). Perceived behavioral control (*β* = −0.029, *p* > 0 0.05) did not have a significant effect on behavior in the adolescents sample.

For college students, the model explained 55.4% of the variance in intention and 30.7% in behavior. Similar to adolescents, perceived behavioral control (PBC; *β* = 0.430) was the strongest antecedent of behavioral intention (BI). BI was a strong predictor of both behavior plan (BP; *β* = 0.643) and behavior (B; *β* = 0.524). However, a key difference emerged: behavior plan (*β* = 0.013, *p* > 0 0.05) and perceived behavioral control (*β* = 0.032, *p* > 0 0.05) did not have a significant effect on behavior in the college student’s sample.

## Discussion

The Theory of Planned Behavior has been extensively applied to understand physical activity, yet a comprehensive quantitative synthesis of its extended model targeting adolescents and college student’s population remain lacking. This study addressed this gap by employing MASEM to integrate findings across studies involving adolescents and college students. Our analysis not only provides meaningful statistical evidence for the relationships in the extended Theory of Planned Behavior (TPB) model but also elucidates the moderating effects of key demographic, methodological, and cultural factors, thereby deepening the theoretical and empirical understanding of physical activity determinants in adolescents and college students. Firstly, pairwise meta-analysis revealed moderate to strong average correlations among all constructs within the extended TPB framework. However, given that *I*^2^ values often exceeded 90% (Table 2), these pooled estimates represent average associations across highly heterogeneous populations, measurement instruments, and study designs, and should therefore be interpreted with caution. This underscores the paramount importance of non-volitional factors, particularly an individual’s sense of control, in forming the intention towards physical activity among adolescents and college student’s people.

Secondly, the MASEM results provide provisional support for the nomological validity of the extended TPB model, though the fit indices were mixed (overall: CFI = 0.944, AGFI = 0.894). All hypothesized paths had statistical significance in the pooled average, and the model accounted for a substantial proportion of the variation in behavioral intention (54.7%) and behavior (29.2%). However, given the extreme heterogeneity observed in the input correlations (*I*^2^ often >90%), these path coefficients represent average structural relationships across heterogeneous populations, instruments, cultures, and study contexts rather than stable, context-invariant estimates. Moreover, these fit statistics should be interpreted in light of the two-stage estimation procedure, which relies on a harmonic mean sample size and pooled correlations rather than raw individual-level data; accordingly, the model is best viewed as approximating the theoretical structure rather than providing definitive confirmation. Notably, perceived behavioral control exhibited the strongest average statistical association with intention among the three TPB antecedents in this cross-study synthesis.

Notably, perceived behavioral control exhibited the strongest statistical association with intention among the three TPB antecedents. However, this finding reflects cross-sectional associations rather than causal effects, and the strongest statistical predictor does not necessarily represent the most effective intervention target. This pattern of results, where PBC dominates the prediction of intention, is consistent with previous extended TPB research (24, 47) and significantly enhances the explanatory power of adolescent moderate to high intensity physical activity intentions (48). Furthermore, the introduction of the behavior plan constructs proved valuable. The strong path from intention to plan, and the significant (though moderate) path from plan to behavior, suggest that plan may be an important mechanism in the process linking motivation with subsequent behavior. A pivotal and intriguing finding of this study is the non-significant direct effect of PBC on physical activity behavior. This result aligns with some previous work (49, 50), but contradicts others (19, 51, 52). It is important to emphasize that the present meta-analysis did not directly test the mechanisms underlying this null association; the following interpretations are therefore speculative and derived from prior theoretical and empirical literature rather than from the current data. According to Ajzen (12) theory, PBC can directly influence behavior only when it accurately reflects actual control. Drawing on this theoretical framework and related literature, we speculate that for adolescents and college students, the transition from perceived control to action may be disrupted by various factors such as overly ambitious goal-setting or an unrealistic assessment of the challenges of physical activity. When goals are set too high, the perceived difficulties (i.e., a sense of self-control) may not facilitate but rather hinder behavioral achievement, as the individual’s control perception does not align with their actual resources and opportunities available to them.

One hypothetical explanation, extrapolated from the broader literature, is that this gap between perception and reality is largely constrained by external environmental factors. For instance, scholars such as Dong et al. (53) found that residents’ perception of climate risks, particularly concerns about heat risks, significantly influences their level of outdoor physical activities. By extension, one might hypothesize that even when adolescents possess a strong sense of control (high PBC), adverse environmental conditions (such as high temperatures or air pollution) or negative perceptions of environmental risks may still act as potent practical barriers to control. If so, these factors could weaken the predictive power of PBC on behavior, potentially contributing to the non-significance of the direct path between PBC and behavior. However, these proposed mechanisms remain untested in the present synthesis and should be treated as hypotheses for future research rather than as conclusions supported by the current meta-analytic data.

The statistical mediating role of intention and the sequential statistical dependency of plan were supported by Bootstrap analysis. These cross-sectional findings describe shared variance among variables but do not establish causal mediation. The significant indirect effects of attitude, subjective norms, and PBC on behavior through intention are consistent with the core tenets of the Theory of Planned Behavior and existing research findings (23, 54, 55). This confirms that these antecedent variables primarily operate by shaping motivational intentions. Moreover, the significant chain mediation effect from the three TPB antecedent variables to intention, then to plan, and finally to behavior, provides a more nuanced understanding of the volitional process. This finding aligns with the proposition that for behaviors not entirely under volitional control, their execution is not only influenced by intention, but also constrained by actual control conditions such as opportunities and resources (12). Behavior plan may serve as concrete strategies to enhance control over these conditions and strengthen the correlation between intention and behavior.

This process of explaining behavior through mediating variables is prevalent in behavioral research. For instance, Ma et al. (56) found in their study on college students’ career exploration that perceived organizational support ultimately influenced exploratory behavior by enhancing individuals’ career adaptability—a key self-regulation resource. This finding provides compelling corroboration for understanding how psychological resources, such as plan ability, can serve as mediating mechanisms that transform motivational antecedents into behavioral outcomes. The findings of this study also underscore the existence of the “intention-behavior gap.” As Sun et al. (57) noted in their exploration of altering consumer inertia purchasing decisions, even when individuals form intentions to change the status quo, entrenched habits and status quo bias may still prevent these intentions from transforming into actual behavior. In this study, behavior plan appears to function as an explanatory process that helps account for the statistical association between intention and behavior. Functioning as a volitional process in our data, behavior plan was positively associated with both intentions and behavior reports, suggesting it may reflect one pathway through which intentions and behaviors are linked. However, because the included studies are largely cross-sectional, we cannot determine whether behavior plan temporally precedes behavior or merely covaries with it, and we therefore refrain from inferring that behavior plan causally mediates the intention–behavior relationship.

Finally, the moderation analysis revealed that the strength of several key relationships in the model is contingent upon the characteristics of participant subgroup. It should be emphasized that subgroup comparisons with fewer than three studies (*k* < 3) are purely descriptive, and no reliable statistical inference can be drawn from these exploratory results. Descriptive comparisons suggested that the effect of attitude on behavior plan appeared stronger in college students than in adolescents (college students: *r* = 0.309 vs. adolescents: *r* = 0.048); however, because the adolescent subgroup comprised only a single study (*k* = 1), this result is purely exploratory and cannot be interpreted as a reliable moderator effect. The effects of attitude on behavior (college students: *r* = 0.295 vs. adolescents: *r* = 0.191, *p* = 0.075) and intention on behavior (college students: *r* = 0.471 vs. adolescents: *r* = 0.351, *p* = 0.057) showed comparable directional trends, though neither reached conventional statistical significance. This finding affirms that demographic factors can moderate the predictive pathways of TPB (12, 58). The weaker intention-behavior consistency among adolescents (*r* = 0.351) compared to college students (*r* = 0.471) could be attributed to the fact that adolescent behavior is less autonomously controlled, often being subject to parental influence and structured environments (19). This group difference reveals the complex interplay between external environments and socio-psychological factors. The overall pattern suggests that the standard TPB model may not fully capture the behavioral dynamics of younger adolescents, potentially neglecting critical factors from other theories, such as social and environmental support (59) or health perceptions (60).

Cil and Eser (61) examined 1,003 Turkish adolescents within a mixed TPB/SCT framework and found that the model explained approximately 60% of intention variance and 45% of behavior variance, though dropping to 28% for behavior among socioeconomically disadvantaged female adolescents. Their results confirmed that attitude (*β* = 0.40) and PBC (*β* = 0.37) were the strongest predictors of intention, while intention strongly predicted behavior (*β* = 0.45). Critically, peer support (*β* = 0.24) was approximately twice as strong as parental support (*β* = 0.12) in promoting behavior, whereas barriers exerted a significant negative effect (*β* = −0.10). These findings corroborate our moderation results regarding the role of subjective norms and social influences, and further highlight that socioeconomic disparities may attenuate the translation of intentions into behavior, particularly through weakened PBC effects among disadvantaged adolescents. This underscores the need for future TPB extensions to explicitly integrate social support dimensions and socioeconomic contexts alongside behavior plan.

The substantial heterogeneity observed in this meta-analysis (with I^2^ values often exceeding 90%) warranted a comprehensive exploration of potential moderators. Beyond the originally examined group types (adolescents and college students), we investigated the influence of cultural background, physical activity measurement method, study design, and gender composition. The results indicated that physical activity measurement method may influence several TPB pathways, with self-report studies tending to yield stronger effect sizes than objective (accelerometer) studies across multiple paths—including attitude–intention (*p* = 0.001), intention–behavior (*p* = 0.001), and subjective norms–behavior (*p* < 0.001). However, these comparisons should be interpreted with caution because the objective-measurement subgroups were based on very few studies (k ≤ 2 for most paths), and the observed differences may be confounded by other study characteristics (e.g., sample composition, study design, or accelerometer data processing protocols) rather than attributable solely to measurement method. This pattern may partly reflect common method variance in self-report studies, where both antecedent variables and behavior were assessed via questionnaires; nevertheless, given the limited number of objective studies, we refrain from drawing firm conclusions about the superiority of one measurement approach over the other.

By contrast, cultural background showed limited explanatory power; although the SN → B association was descriptively stronger in Western (*r* = 0.266) than Eastern (*r* = 0.167) studies, this difference did not reach conventional significance (*p* = 0.077). Overall, the variability in effect sizes across studies may also stem from other unmeasured characteristics, such as socioeconomic status, specific operationalization of TPB constructs, or contextual factors like the built environment. Future research should continue to explore these and other potential moderators to further refine the extended TPB model for adolescents and college student’s physical activity.

## Conclusion

This meta-analysis employed two-stage Meta-analytic Structural Equation Modeling (MASEM) to test the extended Theory of Planned Behavior (TPB) model for physical activity (PA) among adolescents and college students. The findings robustly support the effectiveness of the model, identifying perceived behavioral control (PBC) as the strongest statistical predictor of physical activity intention, though caution is warranted in inferring causality or intervention effectiveness from this cross-sectional evidence. A key contribution is the empirical identification of behavior plan as a significant statistical mediator in the cross-sectional association between behavioral intention and behavior. Whether behavior plan operates as a true causal mediator awaits longitudinal and experimental verification.

The study further reveals the distinctive characteristics of the adolescents and college student’s population. Notably, the absence of a direct effect from PBC to behavior suggests that adolescents and college students’ perceived control may not seamlessly translate into action, potentially due to misaligned goals or resource constraints. Beyond the participant group, the pathways of the model were also moderated by cultural background, measurement methods, and study design, underscoring the contextual sensitivity of the extended TPB framework and indicating that the determinants of physical activity participation vary among different adolescent and college student’s subgroups and methodological conditions.

For practitioners, educators, and health promoters, these insights offer a promising theoretical basis for intervention design. Future intervention research could examine whether a dual-focused strategy—fostering positive attitudes, social norms, and self-efficacy alongside the formation of a concrete and actionable behavior plan—effectively bridges the intention-behavior gap in adolescents and college students physical activity. However, because the present synthesis is based on observational associations, any inference regarding intervention efficacy remains hypothetical and awaits experimental verification.

## Limitations and suggestions

Despite its contributions, this meta-analysis is subject to several limitations that should be considered when interpreting the research findings and that offer avenues for future research.

First, the measurement of the key outcome variable, behavior, was often limited. Many original studies used single-item scales to measure behavior frequency and failed to report its correlations with other TPB constructs. This practice restricted the number of studies and the total sample size available for synthesizing behavior related research, potentially affecting the stability of these estimates.

Second, although the literature search was systematic and comprehensive, it is possible that some relevant studies (such as unpublished dissertations or ongoing work) were missed. This may introduce publication bias. However, the results of the fail-safe N test provide reassurance that a substantial number of null studies would be required to nullify the observed effects in this study (62).

Third, the causal interpretations implied by the model are constrained by the nature of the original studies. The overwhelming majority of included studies relied on cross-sectional surveys, making it impossible to establish firm causal precedence. Furthermore, the use of self-report measurements from a single source at a single point in time may have inflated the observed relationships due to common method variance (63).

Fourth, the MASEM was conducted using a two-stage approach (pooled correlation matrix analyzed via conventional SEM), rather than a full one-stage random-effects MASEM framework. Consequently, the standard errors of the structural path coefficients may be slightly underestimated, and the model fit indices are based on a harmonic mean sample size rather than the full multivariate distribution of study-level data. Future research should consider employing one-stage MASEM (e.g., using the meta SEM package in R) to provide more accurate estimates of standard errors and model fit.

Fifth, the substantial heterogeneity observed across pairwise relationships (*I*^2^ often exceeding 90%) suggests that the pooled correlation coefficients represent broad averages across diverse study contexts rather than precise estimates of a single true effect. While the random-effects model was employed to accommodate this variability, the generalizability of any individual pooled estimate may be limited by differences in population characteristics, measurement instruments, and study designs across the included studies. Consequently, the meta-analytic correlations should be interpreted as average associations within a highly heterogeneous literature, and caution is warranted when applying these estimates to specific subgroups or settings.

Building on these findings and limitations, future research should pursue several promising directions. While this study focused on physical activity, future meta-analyses could investigate whether the extended TPB model functions similarly in other behavioral domains among adolescents and college students, such as dietary habits or screen time. Additionally, although we identified group types (adolescents and college students) as a significant moderator, other potential moderators such as gender, cultural background, and socioeconomic status remain to be further explored. In particular, individual difference variables such as locus of control may play a crucial role. For instance, Chu et al. (64) found that locus of control moderated the relationship between psychological distress and smartphone usage frequency: individuals with an external locus of control increased their usage in distress, while those with an internal locus of control decreased their usage. This suggests that similar dispositional factors could influence the way adolescents and college students translate intentions into physical activity behavior or respond to interventions. Future studies should examine such moderators to refine the extended TPB model. Finally, there is a critical need for more longitudinal and intervention-based studies that can better test the causal hypotheses of the model and further clarify the mechanisms of behavioral change.

## Data Availability

The raw data supporting the conclusions of this article will be made available by the corresponding author upon reasonable request, without undue reservation.
