# Supplementary material for: Behavior plan and the intention–behavior gap in physical activity among adolescents and college students: a systematic review and meta-analysis
Source: Front Public Health. 2026 Sep 24;14:1950356. doi: 10.3389/fpubh.2026.1950356 (PMC13645604; doi:10.3389/fpubh.2026.1950356)
Supplement: Supplementary file 1 [file Data_Sheet_1.zip › Data_Sheet_1/Supplementary/Supplementary Material 1-Characteristics of studies included.docx]

Table S1 Characteristics of studies included in the meta-analysis

| Study | Culture | Sample size | Group type | Female (%) | PA measure type | Study design | BP measure detail |
| --- | --- | --- | --- | --- | --- | --- | --- |
| Liang (2022) | East | 861 | College | 0.465 | Self-report | Cross-sectional | - |
| Feng et al. (2015) - Male | East | 290 | College | 0 | Self-report | Cross-sectional | Planning scale |
| Feng et al. (2015) - Female | East | 330 | College | 100 | Self-report | Cross-sectional | Planning scale |
| Xu et al. (2018) | East | 303 | College | 0.508 | Self-report | Cross-sectional | Planning scale |
| Chen (2017) | East | 516 | College | 0.552 | Self-report | Cross-sectional | - |
| Feng & Mao (2014) | East | 326 | Adolescent | 0.537 | Self-report | Cross-sectional | - |
| Kang & Wang (2016) | East | 353 | Adolescent | 0.504 | Objective | Cross-sectional | - |
| Liu et al. (2022) | East | 1092 | Adolescent | 0.539 | Self-report | Cross-sectional | Action/Coping planning |
| Su et al. (2019) | East | 350 | College | 0.491 | Self-report | Cross-sectional | BPS |
| Su & Xu (2017) | East | 1255 | College | 0.508 | Self-report | Cross-sectional | - |
| Lin & Bai (2014) | East | 134 | College | 0.619 | Self-report | Cross-sectional | - |
| Zhang et al. (2021) | East | 2302 | Adolescent | 0.529 | Self-report | Cross-sectional | - |
| Blanchard et al. (2007) - Black | West | 170 | College | 0.571 | Self-report | Prospective |  |
| Blanchard et al. (2007) - White | West | 180 | College | 0.339 | Self-report | Prospective |  |
| Wang (2011) | West | 517 | College | 0.706 | Self-report | Prospective | - |
| Sicilia et al. (2020) | West | 428 | Adolescent | 0.507 | - | Cross-sectional | - |
| Chen et al. (2022) | East | 1573 | Adolescent | 0.526 | Self-report | Cross-sectional | Implementation Intention |
| Rhodes et al. (2010) | West | 153 | College | 0.74 | Self-report | Prospective | - |
| Luszczynska et al. (2010) - Study 1 | East | 534 | Adolescent | 0.54 | Self-report | Prospective | Coping planning |
| Luszczynska et al. (2010) - Study 2 | West | 620 | Adolescent | 0.62 | Self-report | Prospective | Action planning |
| Cao et al. (2021) | East | 591 | College | 0.575 | Self-report | Prospective | - |
| Wang et al. (2023) | East | 279 | College | 0.369 | Self-report | Prospective | - |
| Shen et al. (2022) | East | 792 | Adolescent | 0.545 | Self-report | Cross-sectional | - |
| Wang & Zheng (2020) | East | 817 | Adolescent | 0.495 | Objective | Cross-sectional | Implementation intention |
| Zhang & Xu (2010) | East | 307 | College | 0.515 | Self-report | Cross-sectional | - |
| Yang et al. (2020) | East | 160 | College | 0.525 | Self-report | Prospective | - |
| Chen et al. (2012) | East | 1456 | College | 0.417 | Self-report | Cross-sectional | - |
| Zhou & Zhou (2009) | East | 596 | College | 0.48 | Self-report | Cross-sectional | - |
| He et al. (2022) | East | 660 | College | 0.5 | Self-report | Cross-sectional | - |
| Wang & Wang (2021) | East | 1219 | College | 0.473 | Self-report | Cross-sectional | Implementation intention |
| Wang & Zhang (2016) | East | 488 | Adolescent | 0.428 | Self-report | Prospective | - |

Note:

Culture: Eastern = China; Western = USA, Canada, Spain, Poland.

Group type：College, Adolescent.

PA measure type: Objective = accelerometer (ActiGraph); Self-report = all questionnaires (e.g., PARS-3, IPAQ, GLTEQ, PAQ-A, self-developed scales).

BP measure detail: BPS = Behavior Planning Scale. Behavioral planning was operationalized as prospective self-regulatory strategies (action plans, coping plans, and implementation intentions) specifying when, where, and how to engage in physical activity.

“—” indicates that the variable was not measured or not applicable.

Study design: Prospective = longitudinal design with follow-up assessment; Cross-sectional = single time-point assessment.
