# Supplementary material for: Behavior plan and the intention–behavior gap in physical activity among adolescents and college students: a systematic review and meta-analysis
Source: Front Public Health. 2026 Sep 24;14:1950356. doi: 10.3389/fpubh.2026.1950356 (PMC13645604; doi:10.3389/fpubh.2026.1950356)
Supplement: Supplementary file 1 [file Data_Sheet_1.zip › Data_Sheet_1/Supplementary/Supplementary Material 2-Methodological quality assessment.docx]

Table S2 Methodological quality assessment using the Newcastle-Ottawa Scale adapted for cross-sectional studies (NOS-xs)

| Study | Selection (0-4) | Comparability (0-2) | Outcome (0-3) | Total score (0-9) | Risk of bias |
| --- | --- | --- | --- | --- | --- |
| Liang (2022) | 3 | 1 | 3 | 7 | low |
| Feng et al. (2015) | 2 | 1 | 3 | 6 | Moderate |
| Xu et al. (2018) | 2 | 1 | 3 | 6 | Moderate |
| Chen (2017) | 2 | 1 | 3 | 6 | Moderate |
| Feng & Mao (2014) | 2 | 0 | 3 | 5 | Moderate |
| Kang & Wang (2016) | 3 | 1 | 3 | 7 | Low |
| Liu et al. (2022) | 3 | 1 | 3 | 7 | Low |
| Su et al. (2019) | 3 | 1 | 3 | 7 | Low |
| Su & Xu (2017) | 3 | 1 | 3 | 7 | Low |
| Lin & Bai (2014) | 2 | 1 | 3 | 6 | Moderate |
| Zhang et al. (2021) | 3 | 1 | 3 | 7 | Low |
| Blanchard et al. (2007) | 3 | 2 | 3 | 8 | Low |
| Wang (2011) | 3 | 2 | 3 | 8 | Low |
| Sicilia et al. (2020) | 3 | 1 | 3 | 7 | Low |
| Chen et al. (2022) | 3 | 1 | 3 | 7 | Low |
| Rhodes et al. (2010) | 3 | 2 | 3 | 8 | Low |
| Luszczynska et al. (2010) | 3 | 2 | 3 | 8 | Low |
| Cao et al. (2021) | 3 | 2 | 3 | 8 | Low |
| Wang et al. (2023) | 3 | 2 | 3 | 8 | Low |
| Shen et al. (2022) | 3 | 1 | 3 | 7 | Low |
| Wang & Zheng (2020) | 3 | 1 | 3 | 7 | Low |
| Zhang & Xu (2010) | 2 | 1 | 3 | 6 | Moderate |
| Yang et al. (2020) | 2 | 1 | 3 | 6 | Moderate |
| Chen et al. (2012) | 3 | 1 | 3 | 7 | Low |
| Zhou & Zhou (2009) | 3 | 1 | 3 | 7 | Low |
| He et al. (2022) | 3 | 1 | 3 | 7 | Low |
| Wang & Wang (2021) | 3 | 1 | 3 | 7 | Low |
| Wang & Zhang (2016) | 3 | 2 | 3 | 8 | Low |

Note:Selection (0–4): Representatives, sample size justification, non-response rate, clear inclusion/exclusion criteria.

Comparability (0–2): Control for important confounding factors (e.g., age, gender) and additional factors.

Outcome (0–3): Valid measurement tool, same method for all participants, appropriate statistical analysis.

Risk of bias classification: Low = 7–9 points; Moderate = 4–6 points; High = 0–3 points.
