# Supplementary material for: Behavior plan and the intention–behavior gap in physical activity among adolescents and college students: a systematic review and meta-analysis
Source: Front Public Health. 2026 Sep 24;14:1950356. doi: 10.3389/fpubh.2026.1950356 (PMC13645604; doi:10.3389/fpubh.2026.1950356)
Supplement: Supplementary file 1 [file Data_Sheet_1.zip › Data_Sheet_1/Supplementary/Supplementary Material 3-Egger's regression test.docx]

Table S3 Egger's regression test for publication bias across all pairwise relationships

| Path | k | Intercept | SE | t | p(2-tailed) |
| --- | --- | --- | --- | --- | --- |
| ATT to B | 21 | -4.321 | 1.797 | 2.405 | 0.027 |
| ATT to BI | 23 | 1.581 | 2.657 | 0.595 | 0.558 |
| ATT to BP | 5 | 12.214 | 9.032 | 1.352 | 0.269 |
| ATT to PBC | 22 | -0.493 | 4.195 | 0.117 | 0.908 |
| ATT to SN | 23 | 0.790 | 4.213 | 0.188 | 0.853 |
| BI to B | 28 | -1.507 | 2.881 | 0.523 | 0.605 |
| BI to BP | 8 | 5.971 | 7.694 | 0.776 | 0.467 |
| PBC to B | 22 | 1.519 | 1.812 | 0.838 | 0.412 |
| PBC to BI | 24 | 0.950 | 2.651 | 0.359 | 0.723 |
| PBC to BP | 6 | -0.579 | 13.077 | 0.044 | 0.967 |
| BP to B | 8 | 1.005 | 5.632 | 0.178 | 0.864 |
| SN to B | 21 | -0.081 | 1.992 | 0.041 | 0.968 |
| SN to BI | 24 | -4.673 | 2.787 | 1.677 | 0.108 |
| SN to BP | 5 | 6.082 | 7.216 | 0.843 | 0.461 |
| SN to PBC | 23 | -1.140 | 3.341 | 0.341 | 0.730 |

Note: k=number of studies; SE=standard error; Egger's test p<0.10 suggests potential publication bias. For the ATT→B relationship, Egger's test was significant (p=0.027), indicating potential publication bias. All other relationships showed no evidence of publication bias (all p>0.05).
